# Effects of journal therapy counseling with anxious pregnant women on their infants’ sleep quality: a randomized controlled clinical trial

**DOI:** 10.1186/s12887-020-02132-7

**Published:** 2020-05-18

**Authors:** Maryam Montazeri, Mojgan Mirghafourvand, Khalil Esmaeilpour, Sakineh Mohammad-Alizadeh-Charandabi, Paria Amiri

**Affiliations:** 1 Department of Midwifery, School of Nursing and Midwifery, Tabriz University of Medical Sciences, Tabriz, Iran; 2 Social Determinants of Health Research Centre, Faculty of Nursing and Midwifery, Tabriz University of Medical Sciences, Tabriz, Iran; 3 Faculty of Education and Psychology, University of Tabriz, Tabriz, Iran; 4 Faculty of Nursing & Midwifery, Tabriz University of Medical Sciences, Tabriz, Iran; 5 School of Nursing and Midwifery, Tabriz University of Medical Science, Tabriz, Iran

**Keywords:** Anxiety, Pregnancy, Journal therapy, Emotional expressiveness, Sleep quality

## Abstract

**Background:**

Sleep is especially important for infants, since it stimulates the development of neural connections in their brains. Psychological stress such as anxiety could affect sleep quality. This study investigated the effects of journal therapy counseling sessions on the infants’ sleep quality based on mothers’ perception (primary outcome), maternal anxiety, infants’ anthropometric and developmental parameters, and the frequency of exclusive breastfeeding (secondary outcomes).

**Methods:**

A total of 70 healthy women with gestational age of 28–31 weeks participated in this randomized controlled trial. The participants were randomly allocated into intervention and control groups using randomized block design. Three in-person journal therapy sessions and three telephone counseling sessions (2 between in-person sessions and 1 one month postpartum) were provided to those in the intervention group, while the control group only received routine care. The Infant Sleep Questionnaire (ISQ), Exclusive Breastfeeding Checklist, and Infant Anthropometric Parameters Checklist were completed at two and four months postpartum. The Beck Anxiety Inventory (BAI) was completed during pregnancy, at the end of the intervention, and at two and four months postpartum, and the Ages and Stages Questionnaire (ASQ) was completed at 4 months postpartum. Data were analyzed using chi-square, independent t-test, ANCOVA and repeated measure ANOVA.

**Results:**

There was no significant difference between the two groups in demographic characteristics and baseline anxiety scores. The mean sleep quality score in infants two months of age (MD: -4.2; 95%CI: − 1.1 to − 7.2; *P* = 0.007) and four months of age (MD: -5.5; 95%CI: − 8.4 to − 2.7; *P* < 0.001) was significantly lower in the intervention group than that of those in the control group. Based on the repeated measure ANOVA results, the mean postpartum anxiety score of mothers in the intervention group was significantly lower than that of those in the control group (AMD: -7.7; 95%CI: − 5.5 to − 10.1; *P* < 0.001). There was no significant difference between the two groups regarding other outcomes including the frequency of exclusive breastfeeding, and anthropometric and developmental parameters (*P* > 0.05).

**Conclusion:**

Journal therapy can decrease mothers’ anxiety and improve the infants’ sleep quality based on their perception. However, further studies are required before drawing any definitive conclusion.

**Trial registration number:**

Iranian Registry of Clinical Trials (IRCT): IRCT20120718010324N45. Date of registration: August 11, 2018. URL: https://en.irct.ir/trial/33211.

## Background

Sleep is a physiological state of relative unconsciousness and inaction of the voluntary muscles, which was first defined as a biological necessity by Gesell and Amatruda in 1941 [1]. Sleep patterns vary in different individuals based on their age, gender, genetics, behavioral, and social factor [2]. Sleep cycles in adults include active (REM) and quiet (NREM) stages [3]. In infants below 6 months of age, there are active, undefined, and quiet stages, respectively [4]. The REM sleep, which is the main stage of sleep in infants, includes closed eyes, active eye movement, irregular shallow breathing, and occasional limb movement. The NREM sleep in infants includes closed eyes, regular deep breathing, and occasional limb movement or sudden panic. The undefined or transitional stage involves some features of active and quiet stages [1].

Sleep is especially importance for infants, because neural connections are formed and some brain areas are developed during sleep [5]. In addition, sleep habits significantly affect infants’ growth [6], awareness, and emotions [7]. Moreover, infants need sufficient sleep for further development of their neurosensory systems, learning centers (hippocampus), pons, brainstem, and midbrain [8]. Sleep is among basic needs of infants, because rapid brain development occurs in early childhood [9]. Sleep quality is generally considered a major determinant of one’s physiological improvement [10], as newborns need 14–17 h of sleep in a 24-h period [2].

Sleep quality refers to an individual’s mental parameters and sleep experience (e.g. feeling relaxed and satisfied after waking up) [11], and various factors such as illness, pain, mental stresses etc. can affect the sleep quality and quantity [12]. In addition, diseases such as colic, iron deficiency anemia and allergies [13], as well as parents’ mental health affect infants’ sleep quality. Meanwhile, parents’ mental health is influenced by their well-being, stressors, stressful life events, low income, anxiety, *etc* [14].

Pregnancy is a highly critical period for developing mental health problems [15], and anxiety disorders are common mental disorders during pregnancy with a prevalence rate of 1 to 26% in low- and middle-income countries [16]. Any person may experience anxiety due to various stressors or environmental pressures [17]; however, this serious psychological factor extremely affects mothers and fetuses during pregnancy [18].

Prenatal anxiety may affect the fetus through specific mechanisms. First, hormones such as catecholamines released due to maternal stress cross the placenta and affect fetal brain development at 12–22 weeks of pregnancy. These hormones also result in umbilical artery contraction which in turn reduces oxygen and nutrients supply to fetus [19]. In addition, maternal anxiety leads to preterm birth, emotional problems, attention deficit hyperactivity disorder (ADHD) symptoms, growth retardation, crying and restlessness, and low mental development in infants [20, 21]. In a study, depression and anxiety disorders were shown to predict infant sleep disorder [22], such that newborns with anxious mothers experience higher restlessness rates [23]. In this regard, 874 mothers between 20 and 34 years of age and their infants participated in a cohort study, and researchers found that maternal psychological distress affects infants sleep quality [24]. In another study, maternal stress was associated with less infant sleep duration at months 4 and 5 [25]. Also, In a prospective longitudinal study performed on primiparous and multiparous women (*n* = 306), the results showed that 10% of excessive infant crying and 12.2% infant sleeping problems were related to maternal anxiety and depression problems [26].

Several therapeutic methods have been developed to improve infants sleep quality. Examples include aromatherapy [27], sleep management training [28], and relaxation and anxiety reduction in parents [29]. In addition, medication, psychotherapy, counseling, and journal therapy, etc. are used for anxiety treatment [30, 31]. Journal therapy is the art of expressing emotions through writing. This counseling approach has positive effects on physical and mental health and overall physiological functioning of individuals [32]. It is used to control post-traumatic stress disorder (PTSD) and schizophrenia, revive memory, reduce pain, develop creativity, and treat acute and chronic anxiety disorders, *etc* [33, 34].

Poor sleep quality has negative impact on infants’ growth and weight gain, and may lead to various behavioral and learning problems [6, 10]. High parental anxiety and mental health problems can disrupt infants sleep [14]. In addition, the authors found no study on controlling maternal anxiety and its impact on infants sleep quality. So, they designed this study to investigate the effect of journal therapy counseling sessions on the infants’ sleep quality based on mothers’ perception.

## Methods

### Study design and participants

The present randomized controlled trial with two parallel groups was conducted between August 2018 and April 2019. The study population consisted of all pregnant women visiting Tabriz Health Centers in Iran.

Inclusion criteria were women in their first or second pregnancy with a gestational age of 28–31 weeks, a moderate anxiety level (based on BAI), and at least a high school diploma. Exclusion criteria included suffering from any mental illness, taking psychiatric drugs, using narcotics and cigarettes (self-reported addiction), having a high-risk pregnancy and high stress and anxiety levels (due to factors such as diabetes, cancer, hypertension, kidney diseases, epilepsy, drug or alcohol addiction, multiple pregnancy, personal or family history of preterm birth or giving birth to an infant with a birth defect), having no intention to take care of the newborn after birth for any reason (e.g. divorce, surrogacy), and a history of giving birth to a child with major physical or mental health problems.

Sample size was calculated using G-Power software based on the results of study by Cronin et al. [35]. It was considered as 38, with regard to the largest standard deviation of infants sleep sub-domains, m_1_ = 20.2 (pre-intervention sleep score), by a default 35% reduction in the mean post-intervention sleep score (m_2_ = 13.13), SD_1_ = SD_2_ = 12.12, α = 0.05, and Power = 80%. The final sample size was 35 considering a loss to follow-up of 10%.

#### Sampling

The sampling was started after obtaining the approval of Ethics Committee of Tabriz University of Medical Sciences (Code: IR.TBZMED.REC.1397.408), as well as permission from the authorities of Tabriz Health Centers, and registering the study at Iranian Registry of Clinical Trials (Code: IRCT20120718010324N45). There are 80 health centers in Tabriz city. Participants were selected from the most crowded health centers in various areas with different socio-economic classes. The author visited the selected centers and obtained data on mothers at the gestational age of 28–31 weeks using the integrated health system (IHS), known as “SIB System”. Then, she called eligible women, provided them with a brief description of the research objective, and asked them to participate in the study. In the first in-person session, eligible women were examined for other exclusion criteria including the Beck Anxiety Inventory, and those with mild and severe anxiety were excluded. Those with moderate anxiety (scores from 16 to 25) completed informed consent forms and the demographic questionnaire. Mothers with severe anxiety were sent to psychology centers. The participants were followed up for up to 4 months postpartum. The BAI was completed during pregnancy, at the end of the intervention, and at two and 4 months postpartum. Mothers completed the Infant Sleep Questionnaire (ISQ) and Exclusive Breastfeeding Checklist at two and 4 months postpartum. The birth anthropometric parameters were extracted from birth records and the author used a weight scale and a tape to measure anthropometric parameters of infants at two and 4months postpartum. Mothers also completed the ASQ at 4 months postpartum.

**Fig. 1:**
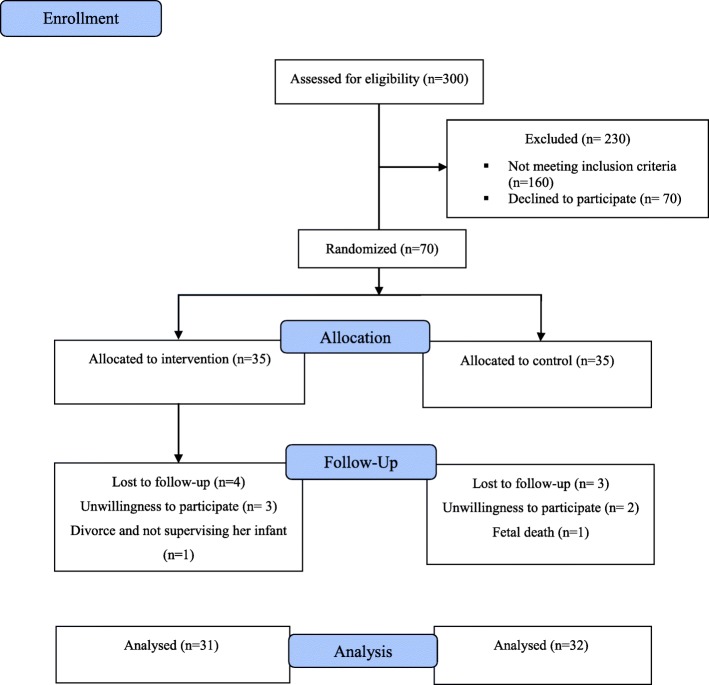
Flow chart of the study

A total of 300 pregnant women were assessed, of whom 70 eligible individuals were enrolled. One hundred sixty individuals were excluded due to not having eligibility criteria (mild to severe anxiety (*n* = 40), poor educational attainments or illiteracy (*n* = 33), and high number of pregnancies (three or more (*n* = 87)) and 70 women declined to participate. Among 70 included participants (35 in each group), three individuals were withdrawn from control group (fetal death (*n* = 1); unwillingness to cooperate (*n* = 2)), and four others were withdrawn from the intervention group (divorce (*n* = 1); unwillingness to cooperate (*n* = 3)) (Fig. 1).

### Randomization

Using randomized block design stratified based on the number of pregnancies (first or second pregnancy) with block sizes of 4 and 6 and a 1:1 allocation ratio, the participants were assigned to the intervention (journal therapy counseling) and control groups. A co-author, other than the data analyzer and the one who selected the participants, assigned them to the groups. To conceal the allocation sequence, the intervention type was written on a piece of paper and placed in opaque envelopes, numbered consecutively.

### Intervention

To reduce anxiety of pregnant women, the first author provided them with three 45–60 min in-person (3–6 person in each group) counseling sessions in weeks 28–31, 32–35 (4 weeks after the first session), and 34–37 (2 weeks after the second session). The first session was held at weeks 28–31 of pregnancy. In this session, the author sought to establish good relationships with participants and gave them a feeling of assurance. Then, she explained the concept of anxiety, relevant factors, negative impacts of anxiety on mothers and their infants’ sleep quality, benefits of sleep, and about how anxiety affects infants sleep. At the end of the session, participants were asked to write down their anxiety factors on a paper in order of prioritize and find potential solutions for each factor and then, hand them over to the author in the next session. The first telephone counseling session was provided by the author 2 weeks later (weeks 30–33) for about 15 min, to follow up and encourage them to carry out their assignments. The second session was held at weeks 32–35. This session was opened with a group discussion on the reported anxiety factors and solutions. Then, the participants were asked to write a story at home about their problems in order to state the causes of their anxiety and identify sources of their problems based on previous tips and discussions. Meanwhile, they were informed that they are free to ask any question. The second telephone session was provided 1 week later, in which the author answered the participants’ questions and examined their ability to manage their anxiety. Mothers were also asked to use the solutions offered in group sessions. The third session was held at weeks 34–37, where previous assignments were reviewed and discussed. Mothers were asked to choose the best solutions suggested by other participants by giving reasons and rewrite their story in the light of the discussed issues. At the end of this session, the author summarized all previous discussions. Finally, at 1 month postpartum, the author called the participants and asked them to employ journal therapy to reduce their anxiety until the end of the study. The control group only received routine pregnancy care during this period.

### Data collection tools

Data were collected using the socio-demographic questionnaire, Beck Anxiety Inventory (BAI), Infant Sleep Questionnaire (ISQ), Ages and Stages Questionnaire (ASQ), Infant Anthropometric Parameters Checklist, and the Exclusive Breastfeeding Checklist.

The socio-demographic questionnaire included questions on mothers and their husbands’ age, educational attainments, occupation, family income, number of pregnancies, type of pregnancy (intended or unintended), etc.

The BAI is a 21-item self-report scale that specifically measures the severity of clinical symptoms of anxiety in adolescents and adults. The items are scored on a four-point Likert scale including not at all (score 0), mild (score 1), moderate (score 2), and severe (score 3). Each item describes one common symptom of anxiety (namely mental, physical, and panic symptoms), and the total score ranges between 0 and 63. The scores are classified as minimal anxiety (0–7), mild anxiety (8–15), moderate anxiety (16–25), and severe anxiety (26–63). High values of content, concurrent, construct, discriminant and factor validity have been obtained for this scale indicating its high efficiency in assessment of anxiety levels. An alpha coefficient of 0.92, a reliability coefficient of 0.75 (with one-week interval), and a correlation between 0.30 and 0.76 have been reported for this scale [36] .

The ISQ is a 10-item questionnaire that assesses infants’ sleep in three domains (going to sleep (3 items), waking up at night (4 items), sleeping in parents’ bed (2 items) and 1 optional item). The total score ranges between 0 and 38, and lower scores indicate higher sleep quality [37]. Mohsenian has examined the validity of the Persian version of this questionnaire, and its reliability has also been confirmed (Cronbach’s alpha coefficient > 0.70) [38].

The ASQ is completed by mothers or caregivers at 4 months postpartum. It easily distinguishes healthy infants from those requiring early interventions. This questionnaire is written in a very simple and straightforward language. Questions (items) are sorted based on their difficulty (from easy to difficult activities). The questionnaire consists of 5 developmental domains, each of which contains 30 items. The items are answered with ‘yes’ (score 10), ‘sometimes’ (score 5), or ‘not yet’ (score 0). Score 10 is given when a child performs the desired activity. The total score ranges between 0 and 300 and the score of each domain ranges from 0 to 60. The final score given to each developmental domain is the summation of all relevant items [39]. We have used cross-cultural adaptation, validation and standardization of Ages and Stages Questionnaire (ASQ) for Iranian Children. Vameghi et al. (2013) assessed the cross-Cultural adaptation, validation and standardization of ASQ in Iranian Children and the results showed that its reliability determined by cronbach’s alpha ranged from 0.76 to 0.86 and the inter-rater reliability was 0.93. The construct validity determined by factor analysis was satisfactory [40].

The Exclusive Breastfeeding Checklist is a 7-item scale that measures the amount of exclusive breastfeeding (without giving additional fluid and solid food to infants) [41]. The infant anthropometric parameters checklist was designed by the research team to record the height, weight, and head circumference of the infants at birth and at 2 and 4 months of age. Weight was measured by a standard and valid scale. A meter was used to measure head circumference and graded ruler to measure height.

### Data analysis

Data were analyzed in SPSS 24. The normality of quantitative data was measured using the Kolmogorov-Smirnov (K-S) test. The chi-square, independent t, and Fisher’s exact tests were used to examine the similarities of the two groups in terms of demographic characteristics. To compare the mean anxiety scores of the two groups before and after the intervention, independent t-test and repeated measure ANOVA (with controlled potential confounding variables) were used, respectively. To compare the mean sleep quality scores of the two groups at two and 4 months postpartum, independent t-test and ANCOVA (with controlled baseline values) were used, respectively. Chi-square test was used to compare the frequency of breastfeeding at two and 4 months postpartum. Independent t-test was used to compare the anthropometric parameters at birth, while ANCOVA (with controlled baseline values) was used to compare these parameters at 2 and 4 months postpartum. Finally, independent t-test was used to compare the developmental domains.

## Results

The mean age of the participants in the both groups was above 27 years. About half of the participants had a high school diploma, most of them were housewives, and their husbands were mainly self-employed. Other socio-demographic characteristics are presented in Table 1.

**Table 1:** Socio-Demographic characteristics of participants in study groups

Variable	Counseling (***n*** = 35) Number (Percent)	Control group (***n*** = 35) Number (Percent)	***P***-value
Woman’s age (Year)	27.5 (5.9)	27.7 (5.8)	0.888^c^
Spouse’s age (Year)	33.3 (5.5)	33.2 (5.4)	0.948^c^
**Woman’s education**			0.847^a^
Under diploma	9 (25.7)	11 (31.4)	
Diploma	16 (45.7)	15 (42.9)	
Academic	10 (28.6)	9 (25.7)	
**Spouse’s education**			0.354^a^
Under diploma	11 (31.4)	16 (45.7)	
Diploma	16 (45.7)	9 (25.7)	
Academic	8 (22.9)	10 (28.6)	
**Job mother**			1.000^b^
Housewife	32 (91.4)	33 (94.3)	
Working at home	1 (2.9)	1 (2.9)	
Working outside	2 (5.7)	1 (2.9)	
**Spouse’s job**			0.542^b^
Unemployed	1 (2.9)	0 (0)	
Worker	4 (11.4)	2 (5.7)	
Employee	6 (17.1)	9 (25.7)	
Self-employment	24 (68.6)	24 (68.6)	
**Sufficiency of income for household expenses**			0.241^a^
Insufficient	6 (17.1)	5 (14.3)	
Somewhat sufficient	24 (68.6)	20 (57.1)	
Sufficient	5 (14.3)	10 (28.6)	
**Type of pregnancy**			1.000^b^
Natural	33 (94.3)	34 (97.1)	
Assisted reproductive techniques	2 (5.7)	1 (2.9)	
**Type of delivery**			0.659^b^
NVD^d^	13 (76.5)	13 (86.7)	
C/S^e^	4 (23.5)	2 (13.3)	
**Infant care**			1.000^b^
Yes	34 (97.1)	35 (100)	
No	1 (2.9)	0 (0)	
**Previous child age** (month)^f^	74.2 (36.2)	93.6 (30.4)	0.116^c^
**Life satisfaction**			0.917^a^
Totally satisfied	13 (37.1)	12 (34.3)	
Somewhat satisfied	12 (34.3)	14 (40)	
Not satisfied not dissatisfied	6 (17.1)	4 (11.4)	
Somewhat dissatisfied	2 (5.7)	3 (8.6)	
Absolutely dissatisfied	2 (5.7)	2 (5.7)	
**Family members number**^f^	2.8 (1.0)	2.5 (.7)	0.215^c^

^a^ Chi-square for trend test

^b^ Fisher’s exact test

^c^Independent t-test

^d^ Normal Vaginal Delivery

^e^ Cesarean section

Variables were reported as numbers (%), except for cases ^f^ reported as mean (Standard Deviation)

The mean (SD) sleep quality score in two-month old infants in the intervention group [8.0 (5.5)] was significantly lower than that of those in the control group [12.2 (6.4)] (MD: -4.2; 95% CI: − 7.2 to − 1.1; *P* = 0.007). The mean (SD) sleep quality score of the 4 months of age infants in the intervention group [5.8 (4.3)] was significantly lower than that of those in the control group [11.4 (6.7)] (MD: -5.5; 95% CI: − 8.4 to − 2.7); *P* < 0.001) (Table 2).

**Table 2:** Comparison of the mean score of infant sleep quality after two and four months after birth in counselling and control groups

variable	Counseling group (***n*** = 35) Mean (SD^b^)	Control group (***n*** = 35) Mean (SD^**⁎**^)	MD (95%CI)^c^	***P***-value
**Infant sleep quality (score range: 0–38)**				
Two months	8.0 (5.54)	12.2 (6.5)	-4.2 (−7.2 to −1.2)	0.007^a^
Four months	5.9 (4.32)	11.4 (6.7)	−5.6 (−8.4 to −2.7)	< 0.00^a^

^a^ Independent T-Test

^**b**^ Standard Deviation

^c^ Mean Difference (95% Confidence Interval)

Before the intervention, there was no significant difference between mean (SD) anxiety score of the mothers in the intervention group [19.3 (3.3)] and that of those in the control group [18.5 (2.8)] (*P* = 0.287). Based on the results of repeated measure ANCOVA test (with adjusted baseline values), 6 weeks after the intervention, mean anxiety score of mothers in the intervention group [13.2 (5.2)] was significantly lower than that of those in the control group [19.4 (5.2)] (MD: -6.8; CI 95%: − 9.1 to − 4.5; *P* < 0.001). At two and 4 months postpartum, mean (SD) anxiety of mothers in the intervention group was 12.4 (6.7) and 9.8 (5.4) and that of those in the control group was 18.8 (7.5) and 17.7 (7.3), and the difference was statistically significant (MD: -7.7; 95% CI: − 5.3 to − 10.1; *P* < 0.001) (Table 3).

**Table 3:** Comparison of the mean score for anxiety before and after intervention in counselling and control groups

variable	Counseling group (***n*** = 35) Mean (SD^c^)	Control group (***n*** = 35) Mean (SD^c^)	MD (95%CI)^d^	***P***-value
**Anxiety (score range: 0–60)**				
Before intervention	19.3 (3.3)	18.5 (2.9)	0.8 (−0.7 to 2.0)	0.287^a^
8 weeks after intervention	13.3 (5.4)	19.4 (5.4)	−7.7 (−10.1 to −5.4)	< 0.001^b^
2 months after childbirth	12.4 (6.7)	18.8 (7.6)		
4 months after childbirth	9.9 (5.5)	17.8 (7.4)		

^a^Independent t-test

^b^ Repeated measure ANOVA

^c^Standard Deviation

^d^ Mean Difference (95% Confidence Interval)

At two and 4 months postpartum, the frequency (percentage) of exclusive breastfeeding was 26 (83.9) and 25 (80.6) in the intervention and was 25 (78.1) and 24 (75.0) in control groups, respectively. There was no significant difference between groups in terms of frequency of exclusive breastfeeding at two (*P* = 0.561) and 4 months (*P* = 0.763) postpartum. Other data on breastfeeding are presented in Table 4.

**Table 4:** Comparison of the frequency of lactation after two and four months in counselling and control groups

variable	Counseling group (***n*** = 35) Number (Percent)		Control group (***n*** = 35) Number (Percent)		***P***-value^a^	
	2 months	4 months	2 months	4 months	2 months	4 months
**Beginning time of lactation**					0.441	
Immediately after delivery	17 (54.8)		15 (46.9)			
During 2 h after delivery	7 (22.6)		5 (15.6)			
2 h after delivery	6 (19.4)		6 (18.8)			
Second day	0 (0.0)		3 (9.4)			
Not reminding exact time	1 (3.2)		3 (9.4)			
**Having 8 times lactation during a day**					0.113	1.00
Yes	31 (100.0)	29 (93.5)	28 (87.5)	29 (90.6)		
No	0 (0.0)	2 (6.5)	4 (12.5)	3 (9.4)		
**Lactation equal or more than 10 min each time**					1.00	0.708
Yes	26 (83.9)	28 (90.3)	27 (84.4)	27 (84.4)		
No	5 (16.1)	3 (9.7)	5 (15.6)	5 (15.6)		
**Lactation during night**					0.492	1.00
Yes	31 (100.0)	31 (100)	30 (93.8)	31 (96.9)		
No	0 (0.0)	0 (0.0)	2 (6.3)	1 (3.1)		
**Number of lactation in night**					0.502	0.389
One time	2 (6.5)	7 (22.6)	5 (15.6)	6 (18.8)		
Two times	6 (19.4)	13 (41.9)	3 (9.4)	16 (50.0)		
Three times	14 (45.2)	8 (25.8)	16 (50)	10 (31.3)		
Equal and more than four times	9 (29)	3 (9.7)	8 (25)	0 (0.0)		
**Number of defecation at least two times**					0.732	0.213
Yes	26 (83.9)	8 (25.8)	28 (87.5)	4 (12.5)		
No	5 (16.1)	23 (74.2)	4 (12.5)	28 (87.5)		
**Exclusive breastfeeding**					0.561	0.763
Yes	26 (83.9)	25 (80.6)	25 (78.1)	24 (75.0)		
No	5 (16.1)	6 (19.4)	7 (21.9)	8 (25.0)		

^a^ Chi-Square Test

There was no significant difference between groups in terms of birth weight (*P* = 0.331), height (*P* = 0.122) and head circumference (*P* = 0.590) of the infants. Also, at two and 4 months postpartum, there was no significant difference between the intervention and control groups in terms of weight (MD: 189.2; 95% CI: − 131.6 to 514.7; *P* = 0.249), height (MD: 0.9; 95% CI: − 0.6 to 2.3; *P* = 0.236) and head circumference (MD: -0.0; 95% CI: − 0.7 to 0.7; *P* = 0.986) (Table 5).

**Table 5:** Comparison of the infant’s anthropometric and developmental indices in counselling and control groups

variable	Counseling group (***n*** = 35) Mean (SD^**⁎**^)	Control group (***n*** = 35) Mean (SD^c^)	MD (95%CI)^d^	***P***-value
**Weight**				
At birth	3227.5 (472.2)	3111.2 (477.9)	116.3 (− 121.2 to 353.9)	0.331^a^
2 months	5001.6 (758.9)	4644.3 (794.6)	189.2 (− 136.1 to 514.7)	0.249^b^
4 months	6550.9 (842.4)	6342.9 (643.0)		
**Height**				
At birth	49.0 (3.15)	47.6 (3.9)	1.4 (−0.4 to 3.18)	0.122^a^
2 months	54.7 (3.51)	52.5 (5.2)	0.9 (−0.6 to 2.3)	0.236^b^
4 months	60.8 (4.32)	58.3 (5.5)		
**Head circumference**				
At birth	35.0 (1.53)	35.2 (1.5)	−0.2 (− 0.9 to 0.6)	0.590^a^
2 months	37.5 (1.34)	37.6 (1.5)	−0.0 (− 0.7 to 0.7)	0.986^b^
4 months	39.9 (1.62)	40.0 (2.0)		
**Developmental indices at 4 months**				
Communication	58.5 (3.7)	56.2 (8.2)	2.2 (−0.9 to 5.5)	^a^0.158
Gross motor	57.1 (6.0)	56.4 (7.2)	0.7 (−2.6 to 4.0)	^a^0.682
Fine motor	59.3 (1.7)	57.6 (6.4)	1.7 (0.7 to 4.1)	^a^0.160
Problem solving	59.5 (1.9)	58.9 (3.9)	0.6 (−0.9 to 2.1)	^a^0.445
Personal Social	57.4 (4.6)	55.9 (8.0)	1.4 (−1.8 to 4.8)	^a^0.377

^a^Independent T-Test

^b^ Repeated measure ANOVA

^c^Standard Deviation

^d^ Mean Difference (95% Confidence Interval)

Finally, no significant difference was found between the two groups in developmental parameters at 4 months of age in the domains of communication (*P* = 0.158.), gross motor skills (*P* = 0.682.), fine motor skills (*P* = 0.160), problem-solving (*P* = 0.445), and personal-social skills (*P* = 0.377) (Table 5).

## Discussion

This study investigated the effects of journal therapy counseling sessions offered to anxious pregnant women visiting Tabriz Health Centers on the infants’ sleep quality based on mothers’ perception (primary outcome), maternal anxiety, infants’ anthropometric and developmental parameters, and the frequency of exclusive breastfeeding (secondary outcomes). The results of this study showed that journal therapy had a significant and positive effect on reducing maternal anxiety during pregnancy and two and 4 months after delivery and also improved infants’ sleep quality based on mothers’ perception at two and 4 months. There was no significant difference between the two groups regarding the frequency of exclusive breastfeeding and anthropometric and developmental parameters of the two- and four-month old infants.

In this study, journal therapy effectively reduced the participants’ anxiety levels. No study was found on the effect of journal therapy on postpartum women; therefore, the results of other studies that have investigated its effect on anxiety levels are discussed in this section. Ali Hassan Zadeh et al. (2012) investigated the effects of journal therapy on anxiety and stress levels of multiple sclerosis patients in Tehran. They divided 80 patients into journal therapy and control groups, and asked those in the intervention group to take their routine medical care, and write about their negative emotions and feelings 30 min a day for four consecutive weeks. Patients in the control group only received routine medical care. Based on the results, expressing emotions via writing significantly reduced anxiety levels in the intervention group compared to those in the control group [42]. Niles et al. (2013) conducted a study entitled “The Effects of Expressive Writing on Psychological and Physical Health” in California. They assigned 116 individuals to expressive writing (*n* = 59) and control (*n* = 57) groups. Those in the intervention group were asked to write for 4 days (20 min a day) their deepest feelings of the most traumatic events happened to them in the past 5 years, and the control group was asked to write about how they have spent their time (without expressing their emotions). After a three-month follow-up period, results showed lower anxiety levels in the intervention group than in the control group [43]. The above results comply with the results of the present study. One of the most important causes of anxiety is unawareness or uncertainty about events [44]. In the journal therapy, words were used in a subtle way to transform obscure and unuttered emotions into conscious words. As a result, this type of intervention reduce negative feelings, and control critical life events [42]

Results indicated the journal therapy counseling sessions improved the infants’ sleep quality based on mothers’ perception. Recent studies that have reported an association between maternal anxiety and their perception of infant sleep quality are scarce. The results of a study showed that the mothers with high anxiety, do not have a proper understanding of their child’s sleep and crying [45]. Pathological concerns are characteristics of anxiety disorders and uncontrollability of concerns is one of the most prominent features of anxiety which causes irrelative concerns about herself and her infant [46]. So, it seems that one of the measures for improving of mothers’ perception is treatment of their anxiety. No studies have previously addressed the effect of journal therapy counseling with anxious mothers on their perception of infants’ sleep quality.

In this study, no significant difference was found between the two groups regarding other outcomes such as the infants’ anthropometric and developmental parameters and the frequency of exclusive breastfeeding. However, some studies [47] have reported correlations between anxiety and neonatal outcomes, and some [48, 49] have not reported such correlations. It should be noted that the above studies are observational studies designed to investigate relationships of different levels of anxiety with these outcomes, while the individuals with severe anxiety were excluded from the present study. According to the theory of Helplessness when pregnant women find themselves exposed to anxiety, in order to control their own potential problems that may affect their offspring, they try to be more careful of the fetus and this increases the anthropometric and other developmental indices of infants [50]. This may be a reason for lack of difference between groups in terms of infants’ anthropometric and developmental parameters. However, it is recommended that these outcomes are considered as primary outcomes in another study on mothers with severe anxiety.

In this study, the authors adhered to all principles of clinical trial such as random allocation and allocation concealment to prevent selection bias. They also sought to earn all participants’ trust and establish similar relationships with all of them. In addition, all staff at the studied health centers sincerely cooperated with the authors and provided them with necessary files and records. The use of standard questionnaires for measuring anxiety levels and sleep quality was among other strengths of this study. In addition, to prevent any withdrawal, counseling schedules were coordinated with the time the participants visiting health centers for routine checkups; however, this partially changed counseling schedules in some cases. In this study, all outcomes were self-reported. To minimize this limitation, the participants were ensured about confidentiality and anonymity as well as the outcomes were measured in different time points. Also, due to the nature of the intervention, the participants and data collector were not blinded. Finally, infant sleep wasn’t measured objectively and the method used to assess infant sleep was subjective maternal report measures. The available methods to measure sleep in young children include polysomnography, videosomnography, actigraphy and parent-report questionnaires. Among these methods, although polysomnography is considered the gold standard for sleep assessment, however, its use in research is limited due to the extensive equipment as well as it requires the laboratory setting. The videosomnography, actigraphy and questionnaires are the most popular sleep measurement methods in infant sleep researches and can be used in the clinical or home environment. Among these three methods, parent questionnaires about infant’s sleep are used prevalently in the literature, perhaps due to their cost-effective and minimally labor-intensive nature [51]. Therefore, parent-report questionnaires can be appropriate to use in studies where parental perceptions of infant sleep are the main focus. It has been showed that the ISQ used in the present study is an acceptable, valid and reliable method for assessing sleep in the infants [37, 38, 52].

## Conclusion

Results indicate that journal therapy improves the infants’ sleep quality based on mothers’ perception. These positive effects are enhanced over time as one performs regular journal therapy exercises, because expressing negative feelings and emotions through journaling reduces daily stresses and increases focus on positive aspects of life. Therefore, it is claimed that this therapeutic approach would be or seems to be a safe, easy and affordable technique to reduce mothers’ anxiety levels over the critical period of pregnancy, provide them with mental peace, and improve their infants’ sleep quality.

## Data Availability

Datasets used and analyzed during this study are available from the corresponding author on reasonable request.

## Abbreviations

ISQ: Infant sleep questionnaire
BAI: Beck anxiety inventory
ASQ: Ages and stages questionnaire
ANCOVA: Analysis of covariance
ANOVA: Analysis of variance
MD: Mean difference
AMD: Adjusted mean difference
REM: Rapid eye movement
NREM: Non-rapid eye movement
